# Astrocytic YAP prevents the glutamate neurotoxicity by upregulation of EAAT2 expression and promotes the gain of stemness in astrocytes in ischemic stroke mice

**DOI:** 10.1038/s41419-025-07806-7

**Published:** 2025-07-30

**Authors:** Xuan Luo, Zhoule Zhu, Tianwen Zheng, Dongmei Li, Yan Wei, Hui Yang, Hong Yu, Jiaqi Han, Ying Wang, Lipei Wang, Zhihui Huang

**Affiliations:** 1 School of Pharmacy, Hangzhou Normal University, Hangzhou, Zhejiang China; 2 Department of Orthopedics (Spine Surgery), the First Affiliated Hospital of Wenzhou Medical University, Wenzhou, Zhejiang China; 3 Department of Clinical Research Center, Affiliated Hangzhou First People’s Hospital, Westlake University School of Medicine, Hangzhou, Zhejiang China; 4 School of Basic Medical Sciences, Hangzhou Normal University, Hangzhou, Zhejiang China

**Keywords:** Stroke, Stem-cell research

## Abstract

The excessive glutamate-mediated excitotoxicity is a major cause of the neuron death in ischemic stroke (IS). Astrocytic glutamate transporter protein-1 (GLT-1, also named excitatory amino acid transporter 2, EAAT2) is essential for maintaining low extracellular glutamate and preventing glutamate neurotoxicity, while its expression is regulated by Yes-associated protein (YAP) signaling reported by our previous study. Recent studies have shown that ischemic injury of the brain induces the gain of stemness in astrocytes dependent on the de novo DNA methyltransferase DNMT3A, and YAP signaling contributes to DNA methylation remodeling upon mouse embryonic stem cell differentiation. However, it remains unknown the roles of astrocytic YAP signaling in IS and whether it regulates the glutamate-mediated excitotoxicity and the gain of stemness in astrocytes induced by IS. In this study, we found that IS was aggravated in YAP^GFAP^-CKO mice with inhibition of the functional behavioral recovery, larger injury area, more apoptotic neurons and more inflammatory infiltration. Furthermore, YAP deletion in astrocytes impaired the formation of glial scars due to the reduction of astrocytic proliferation, and inhibition of activation and gain of stemness in astrocytes induced by IS. Additionally, the expression of EAAT2 was significantly decreased in the cortical astrocytes of YAP^GFAP^-CKO mice after IS through downregulating β-catenin signaling. Activation of EAAT2 by LDN-212320 partially restored the deficits such as neuronal death and behavioral recovery impairment in YAP^GFAP^-CKO mice after IS. Furthermore, activation of astrocytic YAP signaling by XMU-MP-1 upregulated the EAAT2 expression, and inhibited the loss of neurons, and promoted the gain of stemness in astrocytes and functional recovery of mice in IS. These results identify that astrocytic YAP signaling prevents the glutamate neurotoxicity by upregulating EAAT2 expression and promotes the gain of stemness in astrocytes in IS, which provides a novel drug target for IS treatment.

## Introduction

Stroke is defined as the loss of brain function caused by an obstruction or rupture of a blood vessel [1]. Its high prevalence, rapid onset and severity outcome make stroke the second leading cause of death and the third leading source of disability worldwide [2]. Despite the enormous public health burden, currently approved acute stroke treatments are limited to thrombolytic drugs, most notably tissue-type plasminogen activator (tPA or alteplase) and its genetically modified form tenecteplase, or mechanical extraction of thrombus [1]. However, the above treatment time-window is short and prone to have complications such as brain hemorrhage.

The roles of glutamate in neuronal death after excitotoxicity was discovered in the late 1980s, and changed the entire field of stroke research. The idea is that initial oxygen depletion contributes to the inability of cellular ATP generation and the dysregulation of cell membrane ionic homeostasis [3]. Alterations of ionic homeostasis in some glutamatergic neurons depolarize neuronal membranes and activate voltage-dependent calcium channels, with large inward flow of Ca^2+^ leads to a massive release of neurotransmitters such as glutamate into the synaptic gap and extracellularly. These excesses of glutamate lead the overstimulation of ionotropic glutamate receptors (iGluRs) with intracellular signaling cascades activation directly inducing neuronal death by excitotoxicity [4]. To date, glutamate uptake via the EAAT1-5 is the major molecular pathway of regulating extracellular glutamate concentrations and preventing glutamate excitotoxicity. About 80–90% of synaptic interstitial glutamate is taken up by astrocytic EAAT2, which converts glutamate to glutamine and transports it back to the neuron for conversion of glutamate and the inhibitory neurotransmitter gamma-aminobutyric acid (GABA). Glutamate uptake by astrocytes protects neurons from hyperexcitability and subsequent excitotoxic damage by maintaining extracellular glutamate concentrations below neurotoxic levels [5]. Above all, astrocytic EAAT2 is a key protein mediating glutamate uptake metabolism, which in turn affects neuronal excitotoxicity [6]. However, it remains unclear the molecular mechanism of EAAT2 regulation in astrocytes after IS.

Under pathological or injury-induced conditions, intrinsic factors and environmental cues allow astrocytes to re-enter the cell cycle and differentiate into other neuronal cell types [7]. In response to IS or traumatic injury, some astrocytes in non-neurogenic regions are activated and proliferated [8, 9], with the increased expression of GFAP and re-expression of Nestin/Vimentin [10]. Interestingly, several recent studies have shown that IS injury to brain induces the gain of stemness and neurogenesis in some astrocytes such as striatal astrocytes [8, 11–13]. Although some signaling pathways such as Notch-1 signaling and DNA methylation control stemness and neurogenesis of astrocytes after IS [11–13], the detailed molecular mechanism of stemness in astrocytes after IS remains unclear.

The Hippo pathway is an evolutionarily conserved signaling cascade response that serves as a key regulator of tissue growth and many biological processes, including cell growth and fate determination, organ size control, and regeneration [14, 15]. The Hippo pathway is regulated by several extracellular signals such as G protein-coupled receptor (GPCR) signaling, and its major downstream effectors are YAP and transcriptional co-activator with PDZ-binding motif (TAZ) [16, 17]. YAP is closely associated with the proliferation and differentiation of developing neocortical astrocytes [18–20]. Previous studies have shown that activation of YAP after ischemia/reperfusion (I/R) promotes recovery of the blood-brain barrier (BBB) and thus neuroprotection of the damaged brain [21]. Moreover, nuclear localization of astrocytic YAP exerts neuroprotective effects after cerebral ischemic injury in rats by inhibiting STAT3 signaling [22]. In addition, our previous studies have shown that YAP promotes the expression of EAAT2 in astrocytes through β-catenin signaling, which in turn affects glutamate uptake and metabolism [23]. Recent study has shown that ischemia injury-induced neurogenesis in the striatum depends on the de novo DNA methyltransferase DNMT3A [11]. Interestingly, DNMT3A recruited by YAP/TAZ guides DNA methylation to drive gallbladder cancer metastasis [24], and YAP signaling also contributes to DNA methylation remodeling upon mouse embryonic stem cell differentiation [25]. However, the detailed roles and mechanism of astrocytic YAP signaling in IS are still unclear. Therefore, clarifying whether YAP has the regulatory effect of EAAT2 and stemness control of astrocytes in IS and exploring the related molecular mechanisms are also of great significance for the treatment of IS.

In this study, we established YAP^GFAP^-CKO mice with IS model to examine the roles of astrocytic YAP signaling and whether it regulated glutamate metabolism and stemness of astrocytes in IS, and explore the underlying molecular mechanism. We found that astrocytic YAP prevented the glutamate neurotoxicity by promoting EAAT2 expression through β-catenin signaling and promoted the gain of the stemness of astrocytes after IS, which may provide a novel drug target for the treatment of IS.

## Materials and Methods

### Animals

All experiments were approved by the laboratory animal ethics committee of Hangzhou Normal University (HSD20230102) and were conducted following the Reporting of In Vivo Experiments (ARRIVE) guidelines. Mice were housed at a controlled temperature of 22-25 °C, with a 12 h light-dark cycle with free access to food and water in a Specific Pathogen Free (SPF) facility at Hangzhou Normal University. The relevant regulations of the Laboratory Animal Ethics Committee of Hangzhou Normal University were strictly followed in all animal experiments. YAP^GFAP^-CKO mice were generated by crossing GFAP-Cre transgenic mice (Jackson Laboratory) with the YAP allele (YAP^f/f^) mice, maintained in the background of the C57BL/6 J strain. Only male mice were used in all experiments. The YAP^f/f^ mice were generated as described previously [26]. YAP^f/f^ and YAP^GFAP^-CKO mice were assigned randomly to either the sham or IS group using a lottery box, with 3 or 6 mice per group for most experiments. All sample sizes were estimated prior to selection. To examine the effects of EAAT2 activation in mice after IS, comparable groups were established to receive treatment with either a vehicle or LDN-212320 following IS. To examine the effects of YAP activation in mice after IS, comparable groups were established to receive treatment with either a vehicle or XMU-MP-1 following IS. All evaluations were carried out by unbiased investigators who were unaware of the protocols and the types of mice involved. The data were collected by investigators who were blinded to the experimental design, conditions, and treatments in all experiments.

### Photothrombotic stroke (PTS)

Briefly, mice were injected intraperitoneally with Avertin (2, 2, 2-tribromoethanol, Sigma) in saline (0.9% NaCl) via intraperitoneal injection of general anesthetic (0.1 mL/g) prior to surgery. For caudal intravenous administration, Rose Bengal (40 mg/kg body weight, Sigma) was dissolved in saline. The window of the stroke model was placed 2.0 mm to the left of the sagittal suture and 0.5 mm posterior to the coronal suture, and the exposed skull was polished with a high-speed cranial drill, preserving the intact dura mater. The mouse skull was exposed to a green light (532 nm) from a laser generator with a 2 mm aperture positioned on the skull over the left motor cortex. The remaining exposed area of the skull was covered with black tape to prevent undesired illumination. The laser was turned on at 15 mW for 6 min to induce PTS through an intact skull [26, 27]. Mice were sutured and allowed to recover on a heating pad before being returned to their home cages. The sham control mice were injected with saline, and the rest of the operation was same as the IS mice.

### Reagents

LDN-212320 (HY-12741, MedChemExpress) was dissolved in saline containing 1% DMSO, 1% polyethylene glycol 400, 0.2% Tween-80, 10% hydroxypropyl-beta-cyclodextrin, and administered at a concentration of 40 mg/kg by intraperitoneal injection for seven consecutive days [23]. XMU-MP-1 (HY-100526, MedChemExpress) was dissolved in DMSO and injected intraperitoneally at a concentration of 1 mg/kg for four consecutive days before IS. Three consecutive days of injections were started the day after IS [28].

### Western blot

The animals were euthanized and the tissues from the cortex were harvested. Proteins were extracted by incubation on a shaker at 4 °C for 30 min using a lysis buffer containing ice-cold RIPA buffer (P0013B, Beyotime), 100 mM PMSF and 100 mM PIC. The quantification of the proteins was performed by the BCA method (23225, Thermo Fisher Scientific). The proteins were separated on an 8%, 10% or 12% SDS-PAGE gel and then transferred to a nitrocellulose membrane by means of wet transfer. The membranes were then blocked with 5% skim milk and incubated overnight at 4 °C with specific primary antibodies. The following primary antibodies were used in this study: rabbit anti-YAP (1:1, 000, #14074, CST), rat anti-EAAT2 (1: 1, 000, OB-PRT026, Oasis), rabbit anti-β-catenin (1:1, 000, #8480S, CST), rabbit anti-Bax (1:5, 000, ET1603-34, Huabio), rabbit anti-Bcl-2 (1:2, 000, ab182858, Abcam), rabbit anti-IL-1β (1:500, GB11113, Servicebio), rabbit anti-TNF-α (1:400, GB11188, Servicebio). The loading control was detected together with the experimental samples using mouse anti-β-actin (1:10, 000, EM21002, Huabio) or rabbit anti-β-tubulin (1:10, 000, #ET1602-4, Huabio) antibodies. Blots were washed three times with 0.1% Tween-20 in TBST and incubated with appropriate secondary antibodies (Goat anti-rabbit HRP, 1: 10, 000, #FDR007, Fdbio; Anti-mouse IgG HRP, 1: 10, 000, #A9044, Sigma; Goat anti-rat IgG, HRP, 1:5, 000, #RT-HRP, Oasis) for 1 h at room temperature. Protein bands were detected using ECL detection kit (#FD8020, Fdbio science) by imaging system (GelViev 6000Plus, BLT). The densities of the bands were analyzed with the Image J software.

### Histological analysis and immunofluorescence

After mice were anesthetized with tribromoethanol, mice were perfused with 1× phosphate buffer solution (PBS) and 4% paraformaldehyde (PFA) through heart. The brain was then removed, fixed in 4% PFA overnight, and transferred sequentially to 20% and 30% sucrose solutions for dehydration in a density gradient. Cryosections (20 μm) were prepared using a cryostat microtome (Thermo Fisher Scientific), and immunofluorescent staining was performed as followed. Following three washes in PBS, cryosections were fixed in 4% PFA for 30 min followed by incubation with 5% BSA and 0.3% Triton X-100 for 1 h. Cerebral sections were incubated with multiple primary antibodies overnight at 4 °C and rinsed three times with PBS, followed by incubation with appropriate secondary antibodies and 4’, 6’-diamidino-2-phenylindole dihydrochloride (DAPI) (1:1, 000, D9542, Sigma) in 5% BSA for 1 h at room temperature. Primary antibodies included: mouse anti-GFAP (1:500, MAB360, Merck), rabbit anti-YAP (1:200, #14074, CST), mouse anti-NeuN (1:500, E4M5P, CST), rabbit anti-Iba1 (1:100, ab178847, Abcam), rabbit anti-CD45 (1:500, ab10558, Abcam), rabbit anti-CD206 (1:500, #24595, CST), rabbit anti-ALDH1L1 (1:200, OB-PRB001-01, Oasis), chicken anti-Nestin (1:500, NB100-1604, Novus), rabbit anti-Vimentin (1:250, ab92547, Abcam), rabbit anti-S100β (1:200, OB-PRB050-01, Oasis), rabbit anti-PH3 (1:500, #06-570, Merck), rabbit anti-BLBP (1:200, ab279649, Abcam), rabbit anti-Sox2 (1:200, OB-PRB110-01, Oasis), rabbit anti-Sox9 (1:500, OB-PRB049-01, Oasis), rat anti-EAAT2 (1:500, OB-PRT026, Oasis), rabbit anti-β-catenin (1:100, #8480S, CST), rabbit anti-cleaved-caspase 3 (1:400, #9661, CST). Fluorescent secondary antibodies included: goat anti-mouse Alexa Fluor 546 (1:1, 000, A-11030, Invitrogen), goat anti-rabbit Alexa Fluor 488 (1:1, 000, A-11008, Invitrogen), goat anti-chicken Alexa Fluor 633 (1:1, 000, A-21103, Invitrogen), goat anti-Rat Alexa Fluor 647 (1:1, 000, G-RT647, Oasis). Nuclear was stained with 2 ng/mL DAPI. Finally, images of all groups were acquired using a fluorescence microscope (VS200, Olympus) and a confocal microscope (Nikon AX) with the same parameters and analyzed using Adobe Photoshop and ImageJ software.

### Nissl staining

After the mice were perfused with 0.1 mol/L PBS followed by 4% PFA, the brain was immersed in 4% PFA for 24 h and then transferred to a 30% sucrose solution until it was submerged. The cerebrum was then sectioned transversely and horizontally at 20 μm using a cryostat microtome (Thermo Fisher Scientific). After incubation with 0.1% cresyl violet for 5 min at room temperature, rinsing in two series of distilled water followed by 95% ethanol, dehydration in 100% ethanol, and clearing in xylene, the sections were cover-slipped. Images were captured with a microscope (VS200, Olympus). The quantitative analysis of the histological staining and fluorescence was performed by Image J software.

### Infarct volume by TTC method

The obtained brain tissues were placed in a -80 °C refrigerator with rapid freezing for 5 min, then placed on a pre-cooled brain sectioning mold and sectioned at a thickness of 2 mm. The obtained brain sections were placed in 1% 2, 3, 5-Triphenyltetrazolium chloride (TTC) solution and incubated at 37 °C for 25 min. The staining was stopped when the brain tissues turned bright red, and photographed. The size of the infarcted part and whole brain area of each section were measured separately with Image J, and the infarct volume ratio was calculated as the cumulative size of the infarct on the affected side/total area of brain tissue × 100%.

### Laser speckle contrast imaging

Laser speckle contrast imaging (LSCI) was used to evaluate cerebral blood flow (CBF) changes after IS [29]. After deep anesthesia, the head was fixed in a stereotaxic frame (RWD Life Science), and the eyes were covered with erythromycin eye ointment. The skull was then exposed through a midline skin incision. Mineral oil was applied to avoid dryness of the skull. LSCI was performed at different time points (before and 3^rd^ day after IS). At each time point, the mice were continuously monitored for 5 min. The site of injury (a 5 mm diameter circle) was selected as the region of interest (ROI). Blood flow was measured at the ROI and expressed as perfusion units (PUs).

### Neurologic deficit sign score

Sham and IS group mice were placed in an open field, and behavioral evaluations were performed to determine their neurological damage, based on the modified Neurological Severity Score (mNSS) method [30] (a total of 18 points, with larger scores indicating more severe neurological deficits): a) Tail lifting experiment: flexion of the forelimbs (1 point), flexion of the hind limbs (1 point), head deviation from the vertical axis greater than 10° within 30 s (1 point); b) Walking experiment: normal walking (0 points), unable to walk normally (1 point), spinning to the side of light paralysis (2 points), tilting to the side of light paralysis (3 points); c) Sensory experiment: placement experiment (visual and tactile tests) (1 point), proprioceptive experiment (pressing the mice’s paw toward the edge of the table to stimulate limb muscles) (1 point); d) Balance beam experiment: balanced and stable posture (0 points), clinging to the edge of the balance beam (1 point), clinging to the balance beam with one limb slipping (2 points), clinging to the balance beam with both limbs dangling or rotating from the balance beam (greater than 60 s) (3 points), attempting to balance on the balance beam but falling (greater than 40 s) (4 points), attempting to balance on the balance beam but falling (greater than 20 s) (5 points), falling, not attempt to balance on balance beam (less than 20 s) (6 points); e) Reflexes and abnormal activity: auricular reflex (shaking head on contact with external auditory canal) (1 point), corneal reflex (blinking on touching cornea with cotton wool) (1 point), startle reflex (motor reflex or screaming on hearing a sudden sound) (1 point), epilepsy, myoclonus, dystonia (1 point).

### Grid tests

Mice were allowed to walk on a 35 cm × 30 cm wire grid, with an 11 mm-square mesh fixed 40 cm above the ground frame for 5 min [31]. A camera was placed beneath the grid to record video for assessing errors of the left forelimb during the first 100 steps.

### Cylinder tests

The mice were placed in a clean and transparent cylinder (20 cm in diameter and 40 cm in height) and were videotaped for 3 min from the time they entered the cylinder, and the number of times that the affected and healthy forepaws touched the wall of the cylinder was recorded by the video [31]. Forelimb asymmetry index for mice was calculated as: (number of Left contacts – number of Right contacts) / (number of Left contacts + number of Right contacts +number of both paws contacts).

### Corner tests

The mice were placed into a 30° corner [32], to ensure that these mice had to turn to leave the corner, and the number of turns to the left and to the right was recorded, respectively (repeat the test 10 times, with a 30-second interval between each test). Corner turning score for mice was calculated as: Number of right turns / (Number of left turns + Number of right turns) × 100%.

### Rotarod tests

The rotational speed was gradually increased from 4 rpm to 40 rpm over a period of 5 min, and the mice were acclimatized and trained to perform three repetitions of the experiment, each of which lasted 5 min, with a 30 min interval between experiments, and the average time of the mice fell from the rotating rod was calculated for the purpose of analyzing the neurological function [33].

### Open field tests

The mice were placed in the center of the open field. The video recording function of the software was turned on, and the activities of the mice in the open field were recorded for 15 min, and the total distance of the mice’s movements within the same period of time was calculated to compare the neurological deficits of the mice [34].

### Statistical analysis

All results were statistically analyzed with data from at least three mice per group, and the data were expressed as mean ± SEM of no less than 3 independent experiments. Statistical analysis was conducted using GraphPad Prism 8.0 and Image J software. Unpaired *t*-test analysis, one-way or two-way ANOVA with Tukey’s multiple-comparison test were employed. Statistical significance was considered at *P* < 0.05. Additional details were provided in the figure legends.

## Results

### YAP was upregulated and translocated into nuclear of astrocytes in mice after IS, and YAP deletion in astrocytes inhibited the functional behavior recovery and aggravated injury in mice after IS

**Fig. 1:**
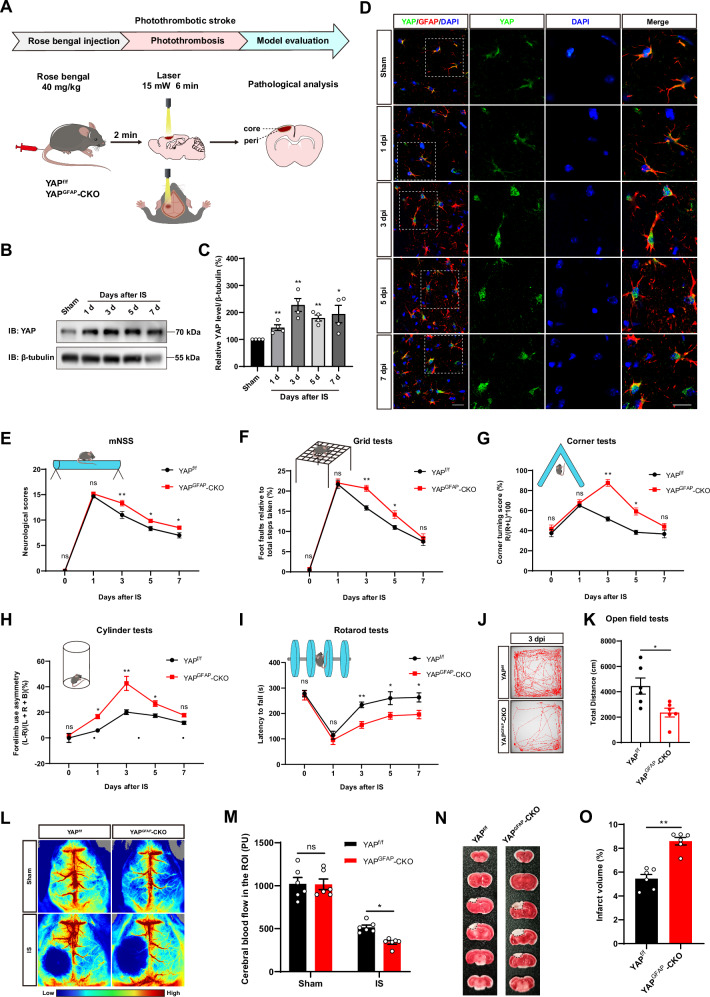
YAP was upregulated and translocated into nuclear of astrocytes in mice after IS, and YAP deletion in astrocytes inhibited the functional behavior recovery and aggravated injury in mice after IS. **A** Schematic diagram of photochemical embolization (PT) modeling: mice were injected with 40 mg/kg rose bengal by tail vein, and a 532 nm fixed-wavelength laser was fixedly irradiated at the bone window with a constant light intensity for 6 min. **B** Western blot detected the expression levels of YAP in brain tissues extracted from the Sham group and the IS group of wild-type mice at 1^st^, 3^rd^, 5^th^ and 7^th^ day after IS. **C** Quantitative analysis of the relative expression level of YAP as shown in (**B**) (normalized to sham, *n* = 4 blots from 4 mice). **D** Double immunostaining of YAP (green) and GFAP (red) in the cortex from the Sham and IS groups of wild type mice at 1^st^, 3^rd^, 5^th^ and 7^th^ day after IS modeling. DAPI was stained for nucleus signal. Scale bars: 20 μm. **E–K** Behavioral analysis of YAP^f/f^ and YAP^GFAP^-CKO mice at indicated days after IS by mNSS (**E**), grid tests (**F**), corner tests (**G**), cylinder tests (**H**), rotarod tests (**I**) and open field tests (**J–K**) (*n* = 6 mice each group). **L** Representative images were taken by laser speckle imaging of regional CBF in the cortical region of YAP^f/f^ and YAP^GFAP^-CKO mice at 3^rd^ day after IS. **M** Quantitative analysis of CBF changes before and after IS as shown in **L** (*n* = 6 mice per group). **N** Representative images of brain tissues of YAP^f/f^ and YAP^GFAP^-CKO mice at 3^rd^ day after IS analyzed by TTC staining. **O** Quantitative analysis of cerebral infarct volume in mice as shown in (**N**) (*n* = 6 mice per group). **C** Data were analyzed by one-way ANOVA with Tukey’s multiple-comparison test. **E–I**, **M** Data were analyzed by two-way ANOVA with Tukey’s multiple-comparison test. **K**, **O** Data were analyzed by unpaired *t*-test analysis. n.s. indicated no statistical difference (*P* > *0.05*); ^*^P < *0.05*, ^**^P < *0.01*, Mean ± SEM.

To explore the function of YAP in IS, we initially investigated its spatiotemporal expression pattern using PTS model of cerebral ischemic (Fig. 1A). Mice were sacrificed at 3^rd^ day following PTS, and the brain sections were then subjected to TTC staining, a commonly used method for observing limited and well-defined cerebral infarction in mice. TTC staining clearly outlined the lesions from the surrounding ischemic core region in the IS mice (Supplemental Data Figure S1A, B), indicating the model was established successfully. Protein samples were collected from the cerebral cortex of mice, and Western blot was utilized to examine the expression of YAP at 1^st^, 3^rd^, 5^th^, 7^th^ days after IS, and it was found that YAP protein was significantly upregulated, and peaked at 3^rd^ day after IS (Fig. 1B, C). Immunostaining showed that YAP was predominantly expressed in the cytoplasm of GFAP^+^ astrocytes in the Sham control mice, but it exhibited the partial nuclear localization at 1^st^ day of IS, and displayed a significant nuclear localization in GFAP^+^ astrocytes from 3^rd^ to 7^th^ day after IS (Fig. 1D). These results suggested that YAP was upregulated and translocated into nuclear of astrocytes following IS, implying the activation of astrocytic YAP signaling in IS.

To further confirm the roles of astrocytic YAP in IS, YAP^GFAP^-CKO mice were generated with a conditional YAP deletion specifically in astrocytes by crossing floxed YAP allele (YAP^f/f^) with GFAP-Cre transgenic mice (Supplemental Data Figure S1C, D). YAP was indeed knockout in most brain regions such as cortex, hippocampus, cerebellum and spinal cords (Supplemental Data Figure S1E, F), as well as the cortical astrocytes (Supplemental Data Figure S1G). Several behavioral tests, including mNSS, grid tests, cylinder tests and rotarod tests, showed no significant differences in locomotor ability and left and right limb asymmetry between YAP^f/f^ mice and YAP^GFAP^-CKO mice (Supplemental Data Figure S1H–K), suggesting that YAP deletion in astrocytes did not affect the locomotor function of mice. Subsequently, we performed IS model in YAP^f/f^ and YAP^GFAP^-CKO mice. Interestingly, as shown in Fig. 1E, YAP^GFAP^-CKO mice exhibited a significant increase in mNSS, compared with that in YAP^f/f^ mice after IS. Meanwhile, we also performed additional functional behavioral tests including the grid tests, corner tests, cylinder tests and rotarod tests. These assays also showed a significant inhibition of behavioral recovery in YAP^GFAP^-CKO mice after IS, especially at 3^rd^ day (Fig. 1F–I), indicating more severe damages of central nervous system (CNS) in YAP^GFAP^-CKO mice. Finally, we conducted the open field tests at 3^rd^ day after IS, and the result showed total distances was significantly decreased in the YAP^GFAP^-CKO mice, which was also consistent with the other behavioral tests (Fig. 1J, K). Therefore, the 3^rd^ day after IS was identified as a critical time point for further studies. We next examined the pathological changes of brain infarct in YAP^GFAP^-CKO mice after IS. Laser scatter imaging showed that CBF was significantly lower at the infarct site of YAP^GFAP^-CKO mice at 3^rd^ day after IS (Fig. 1L, M). TTC staining further showed a larger infarct volume in YAP^GFAP^-CKO mice at 3^rd^ day after IS (Fig. 1N, O). Taken together, these results suggested that YAP deletion in astrocytes inhibited the functional behavior recovery and aggravated injury in mice after IS.

### IS was aggravated with more apoptotic neurons in YAP^GFAP^-CKO mice

**Fig. 2:**
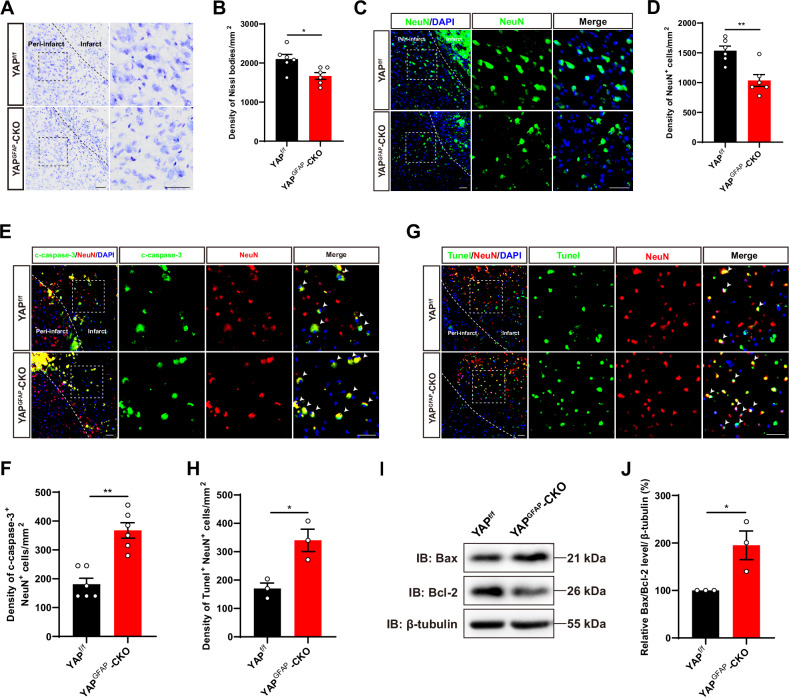
IS was aggravated with more apoptotic neurons in YAP^GFAP^-CKO mice. **A** Representative images of Nissl staining of the cortex of YAP^f/f^ and YAP^GFAP^-CKO mice at 3^rd^ day after IS. **B** Quantification of the density of Nissl bodies as shown in (**A**) (n = 6 mice per group). **C** Immunostaining of NeuN (green) in the cortex of YAP^f/f^ and YAP^GFAP^-CKO mice at 3^rd^ day after IS. **D** Quantification of the density of NeuN^+^ cells as shown in (**C**) (*n* = 6 mice per group). **E** Double immunostaining of c-caspase-3 (green) and NeuN (red) in the cortex of YAP^f/f^ and YAP^GFAP^-CKO mice at 3^rd^ day after IS. **F** Quantification of the density of apoptotic NeuN^+^ cells as shown in (**E**) (*n* = 6 mice per group). **G** Double immunostaining of Tunel (green) and NeuN (red) in the cortex of YAP^f/f^ and YAP^GFAP^-CKO mice at 3^rd^ day after IS. **H** Quantification of the density of apoptotic NeuN^+^ cells as shown in (**G**) (*n* = 3 mice per group). **I** Western blot analysis of Bax and Bcl-2 expression in the cortex of YAP^f/f^ and YAP^GFAP^-CKO mice at 3^rd^ day after IS. **J** Quantification of the relative Bax/Bcl-2 expression level as shown in (**I**) (*n* = 3 mice per group, normalized to YAP^f/f^ control mice). Scale bars: 50 μm. Data were analyzed by unpaired *t*-test analysis (**B**, **D**, **F**, **H**, **J**). ^*^P < *0.05*, ^**^P < *0.01*, Mean ± SEM.

To further elucidate the underlying mechanisms of impaired post-IS behavioral recovery caused by astrocyte-specific YAP deletion, we next examined the loss of neuron in YAP^GFAP^-CKO mice after IS. As expected, the density of Nissl bodies was significantly reduced within the infarct area in YAP^GFAP^-CKO mice (Fig. 2A, B). Simultaneously, we observed a more pronounced loss of neurons at the peri-infarct area in YAP^GFAP^-CKO mice by immunostaining of NeuN (a neuronal marker) (Fig. 2C, D). DNA damage is one of the main features of apoptosis visualized with c-caspase-3 staining. As expected, the c-caspase-3 positive neurons were significantly increased at the peri-infarct in YAP^GFAP^-CKO mice after IS (Fig. 2E, F). Similarly, Tunel-positive neurons were also significantly increased at the peri-infarct of YAP^GFAP^-CKO mice after IS (Fig. 2G, H). In addition, the expression ratio of apoptosis-related protein Bax/Bcl-2 was higher in the infarct area of YAP^GFAP^-CKO mice (Fig. 2I, J). These results suggested that YAP deletion in astrocytes aggravated IS with more apoptotic neurons, indicating the neuroprotective effects of astrocytic YAP signaling in IS.

### IS was aggravated with more inflammatory infiltration in YAP^GFAP^-CKO mice

**Fig. 3:**
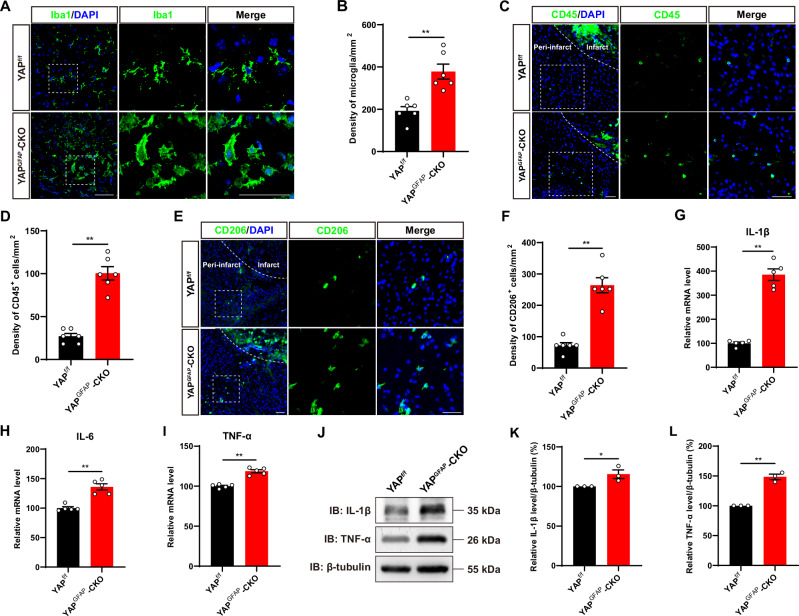
IS was aggravated with more inflammatory infiltration in YAP^GFAP^-CKO mice. **A, C, E** Immunostaining of Iba1 (green) (**A**) or CD45 (green) (**C**) or CD206 (green) (**E**) in the cortex of YAP^f/f^ and YAP^GFAP^-CKO mice at 3^rd^ day after IS. **B**, **D**, **F** Quantification of the density of Ibal^+^ cells (**B**) or CD45^+^ cells (**D**) or CD206^+^ cells (**F**) as shown in (**A**) or (**C**) or (**E**), respectively (*n* = 6 mice per group). **G–I** qPCR analysis showed the relative mRNA level of *IL-1β* (**G**), *IL-6* (**H**) and *TNF-α* (**I**) in YAP^f/f^ and YAP^GFAP^-CKO mice at 3^rd^ day after IS (*n* = 5 mice per group, normalized to YAP^f/f^ control mice). **J** Western blot analysis of IL-1β and TNF-α expression in the cortex of YAP^f/f^ and YAP^GFAP^-CKO mice at 3^rd^ day after IS. **K, L** Quantification of the relative IL-1β (**K**) and TNF-α (**L**) expression level, as shown in (**J**) (*n* = 3 mice per group, normalized to YAP^f/f^ control mice). Scale bars: 50 μm. Data were analyzed by unpaired *t*-test analysis (**B**, **D**, **F–I**, **K**, **L**). ^*^P < *0.05*, ^**^P < *0.01*, Mean ± SEM.

Following acute IS, secondary neuroinflammation exacerbates injury and significantly contributes to neuronal death. Simultaneously, proinflammatory signals from immune mediators rapidly activate resident microglia and influence the infiltration of various inflammatory cells into the ischemic region to exacerbate brain damage [35]. At 3^rd^ day after IS, immunostaining showed that the density of Iba1^+^ cells (activated microglia) was significantly increased at the edge of the infarct in YAP^GFAP^-CKO mice (Fig. 3A, B). Meanwhile, more pronounced morphological changes were observed in the Iba1^+^ microglia cells in YAP^GFAP^-CKO mice. These microglia cells showed the enlarged cell bodies and shorter processes (Fig. 3A). Additionally, we assessed the number of CD45^+^ cells (neutrophil) and CD206^+^ cells (macrophage) by immunostaining after IS. As shown in Fig. 3C–F, the density of both CD45^+^ cells and CD206^+^ cells were significantly increased in YAP^GFAP^-CKO mice after IS, suggesting that the inflammatory infiltration was more severe in YAP^GFAP^-CKO mice after IS. Furthermore, qPCR results showed that the mRNA expression levels of inflammatory factors such as *IL-1β*, *IL-6* and *TNF-α* were significantly upregulated in YAP^GFAP^-CKO mice at 3^rd^ day of IS (Fig. 3G–I). Western blot further confirmed that the IL-1β and TNF-α protein expressions were increased in the infarct area of YAP^GFAP^-CKO mice at 3^rd^ day of IS (Fig. 3J–L). Taken together, these results suggested that YAP deletion in astrocytes aggravated IS with more inflammatory infiltration.

### YAP deletion in astrocytes impaired the formation of glial scars due to the reduction of astrocytic proliferation in mice after IS

**Fig. 4:**
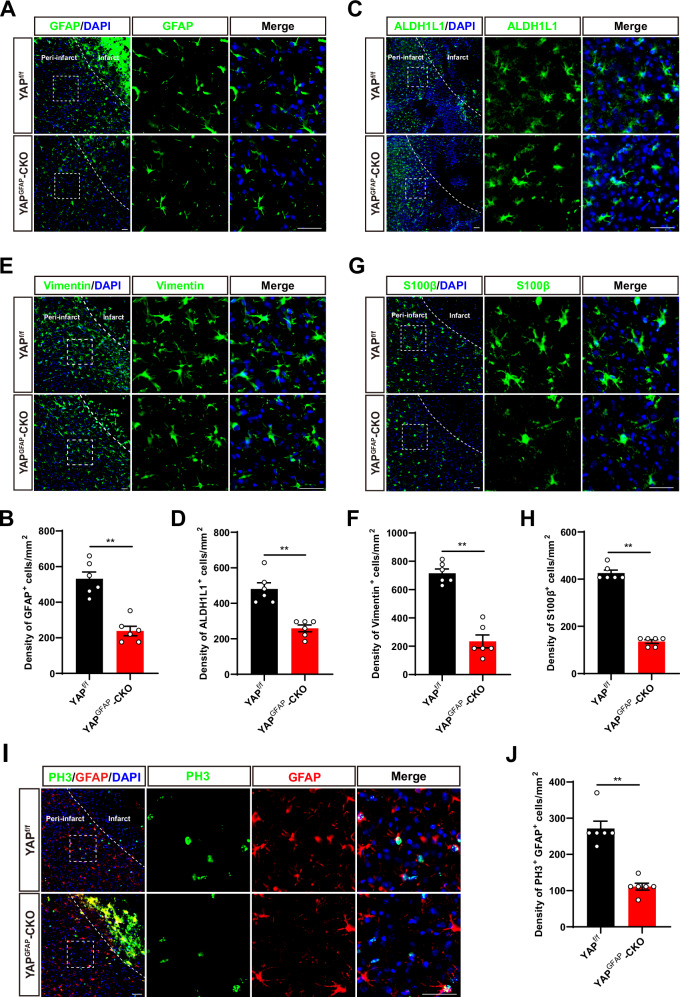
YAP deletion in astrocytes impaired the formation of glial scars due to the reduction of astrocytic proliferation in mice after IS. **A, C, E, G** Immunostaining of GFAP (green) (**A**) or ALDH1L1 (green) (**C**) or Vimentin (green) (**E**) or S100β (green) (**G**) in the cortex of YAP^f/f^ and YAP^GFAP^-CKO mice at 3^rd^ day after IS. **B, D, F, H** Quantification of the density of GFAP^+^ cells (**B**) or ALDH1L1^+^ cells (**D**) or Vimentin^+^ cells (**F**) or S100β^+^ cells (**H**) as shown in (**A**) or (**C**) or (**E**) or (**G**), respectively (*n* = 6 mice per group). **I** Double immunostaining of PH3 (green) and GFAP (red) in the cortex of YAP^f/f^ and YAP^GFAP^-CKO mice at 3^rd^ day after IS. **J** Quantification of the density of PH3^+^ and GFAP^+^ cells as shown in (**I**) (*n* = 6 mice per group). Scale bars: 50 μm. Data were analyzed by unpaired *t*-test analysis (**B, D, F, H, J**). ^**^P < *0.01*, Mean ± SEM.

After IS, reactive astrocyte proliferation limits tissue loss and restores tissue homeostasis and also affects ischemia-induced plasticity and functional recovery [36, 37]. Within a few days of ischemic attack, astrocytes in the peri-infarct region undergo hypertrophy and other cellular processes for forming a neuroglial scar around the damaged area to prevent the spread of infiltrating leukocytes into the surrounding healthy parenchyma [38, 39]. Hence, the alterations of astrocytes and glial scar post IS was the next focus of our investigation. Interestingly, we found that the density of GFAP^+^ cells (a marker of glia scar) was significantly decreased at the peri-infarct area in YAP^GFAP^-CKO mice, compared with that in YAP^f/f^ mice at 3^rd^ day after IS (Fig. 4A, B). In addition, we confirmed this phenomenon by ALDH1L1, a more widely and specifically expressed astrocyte marker. As expected, the density of ALDH1L1^+^ cells at the peri-infarct area was also significantly reduced in YAP^GFAP^-CKO mice after IS (Fig. 4C, D). Vimentin, an intermediate filament protein reflects the migratory capacity of responsive astrocytes to constitute the glial scars [40–42]. As shown in Fig. 4E, F, the density of Vimentin ^+^ cells was also significantly decreased at the peri-infarct area in YAP^GFAP^-CKO mice. S100β is a small calcium-binding protein found primarily on astrocytes as one of the most specific markers for astrocytes, and a potential biomarker for BBB integrity and astrocyte-associated brain injury [43]. Similarly, we also observed a significant decrease in density of S100β^+^ astrocytes at the peri-infarct of YAP^GFAP^-CKO mice after IS (Fig. 4G, H). We next examined whether the impairment of glia scar formation was due to the reduction of astrocytic proliferation in YAP^GFAP^-CKO mice. Indeed, the density of PH3^+^ (a cell marker of proliferation) cells was significantly decreased in GFAP^+^ astrocytes of YAP^GFAP^-CKO mice after IS (Fig. 4I, J). Taken together, above results suggested that YAP deletion in astrocytes impaired the formation of glial scars due to the reduction of astrocytic proliferation after IS.

### YAP deletion in astrocytes suppressed astrocytic activation, and inhibited the gain of stemness in astrocytes induced by IS in mice

**Fig. 5:**
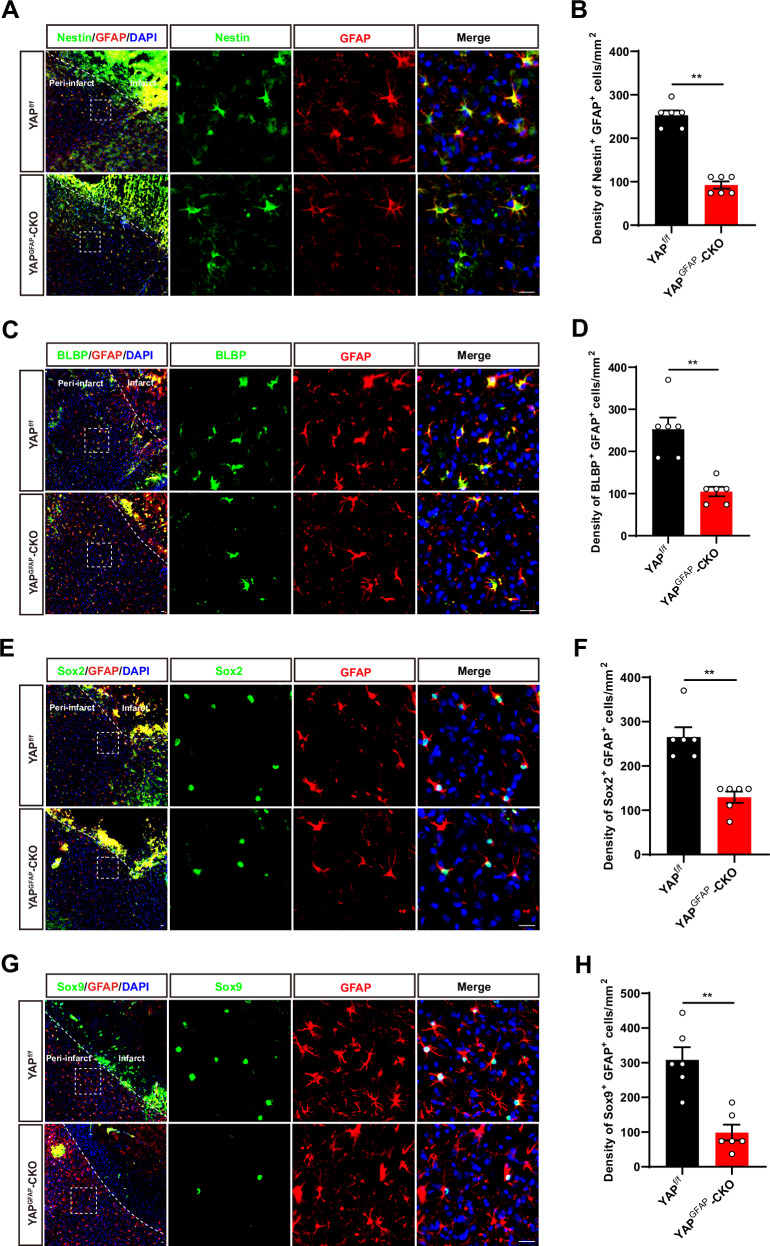
YAP deletion in astrocytes suppressed astrocytic activation, and inhibited the gain of stemness in astrocytes induced by IS in mice. **A** Double immunostaining of Nestin (green) and GFAP (red) in the cortex of YAP^f/f^ and YAP^GFAP^-CKO mice at 3^rd^ day after IS. **B** Quantification of the density of Nestin^+^ and GFAP^+^ cells as shown in (**A**) (*n* = 6 mice per group). **C** Double immunostaining of BLBP (green) and GFAP (red) in the cortex of YAP^f/f^ and YAP^GFAP^-CKO mice at 3^rd^ day after IS. **D** Quantification of the density of BLBP^+^ and GFAP^+^ cells as shown in (**C**) (*n* = 6 mice per group). **E** Double immunostaining of Sox2 (green) and GFAP (red) in the cortex of YAP^f/f^ and YAP^GFAP^-CKO mice at 3^rd^ day after IS. **F** Quantification of the density of Sox2^+^ and GFAP^+^ cells as shown in (**E**) (*n* = 6 mice per group). **G** Double immunostaining of Sox9 (green) and GFAP (red) in the cortex of YAP^f/f^ and YAP^GFAP^-CKO mice at 3^rd^ day after IS. **H** Quantification of the density of Sox9^+^ and GFAP^+^ cells as shown in (**G**) (*n* = 6 mice per group). Scale bars: 20 μm. Data were analyzed by unpaired *t*-test analysis (**B**, **D**, **F**, **H**). ^**^P < *0.01*, Mean ± SEM.

Astrocytes with pathological and injured states not only proliferate, but also enter an intermediate state of neural stemness, where they undergo intense differentiation [7]. Recent studies have shown that ischemic injury to the brain induces the gain of stemness in astrocytes dependent on the de novo DNA methyltransferase DNMT3A [11]. Interestingly, YAP signaling contributes to DNA methylation remodeling upon mouse embryonic stem cell differentiation [25]. Therefore, we next examined whether YAP deletion affected reactivation and stemness of astrocytes induced by IS. Nestin, a neural stem cell marker that was labeled activated astrocytes with neural stemness [44], was performed by immunofluorescence staining co-localized with GFAP. Interestingly, as shown in Fig. 5A, B, the density of GFAP and Nestin double-positive cells was significantly decreased at the peri-infarct area of YAP^GFAP^-CKO mice at 3^rd^ day after IS, and the density of GFAP with BLBP (a marker of astrocyte transition to intermediate state [45]) double-positive cells was also significantly reduced (Fig. 5C, D). Sox2 is a transcription factor belonging to the SRY-box (sex-determining region Y-box) gene family, which is important for neural stem cells, neural development and stemness maintenance [46]. The immunostaining showed that the density of GFAP and Sox2 double positive astrocytes was significantly decreased in YAP^GFAP^-CKO mice (Fig. 5E, F). Sox9 is the major SoxE factor that induces and maintains neural stem cells. In astrocyte precursors, Sox9 is the downstream of Notch signaling and plays a key role in their migration, metabolism and transcriptional regulation [47]. The immunostaining showed that the density of GFAP and Sox9 double positive astrocytes was also significantly decreased in YAP^GFAP^-CKO mice (Fig. 5G, H). Taken together, these results suggested that YAP deletion in astrocytes suppressed the activation of astrocytes and inhibited the gain of stemness in astrocytes at 3^rd^ day after IS.

### The expression of EAAT2 was downregulated in the cortical astrocytes of YAP^GFAP^-CKO mice after IS

**Fig. 6:**
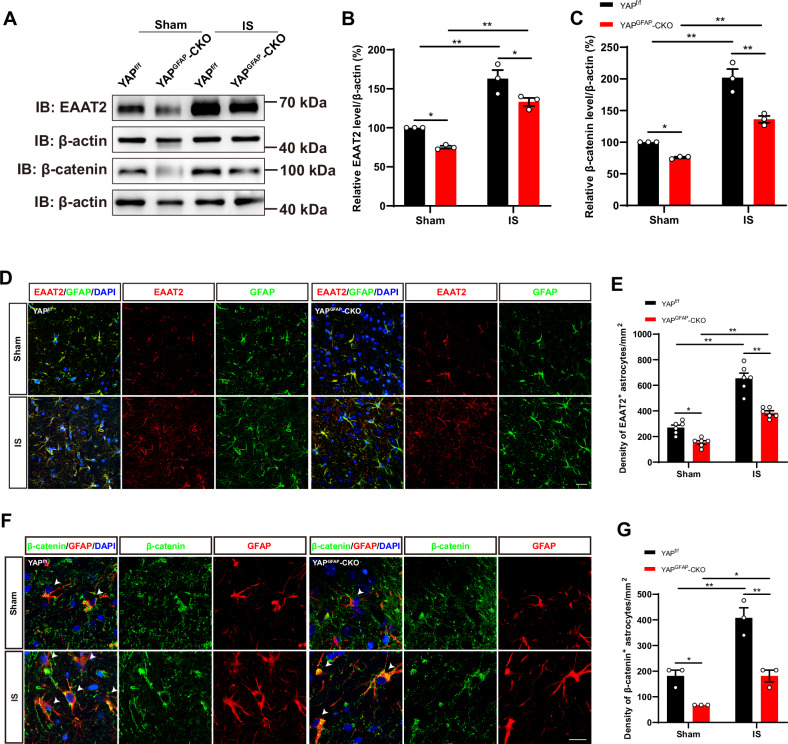
The expression of EAAT2 was downregulated in the cortical astrocytes of YAP^GFAP^-CKO mice after IS. **A** Western blot detected the expression levels of EAAT2 and β-catenin in brain tissues extracted from the Sham group and the IS group of YAP^f/f^ and YAP^GFAP^-CKO mice at 3^rd^ day after IS. **B, C** Quantitative analysis of the relative level of EAAT2 (**B**) and β-catenin (**C**) as shown in (**A**) (*n* = 3 mice per group). **D** Double immunostaining of GFAP (green) and EAAT2 (red) in the cortex of YAP^f/f^ and YAP^GFAP^-CKO mice at 3^rd^ day after IS. **E** Quantification of the density of GFAP^+^ and EAAT2^+^cells as shown in (**D**) (*n* = 6 mice per group). **F** Double immunostaining of β-catenin (green) and GFAP (red) in the cortex of YAP^f/f^ and YAP^GFAP^-CKO mice at 3^rd^ day after IS. **G** Quantification of the density of β-catenin^+^ and GFAP^+^ cells as shown in (**F**) (*n* = 3 mice per group). Scale bars: 20 μm. Data were analyzed by two-way ANOVA with Tukey’s multiple-comparison test. **B**, **C**, **E**, **G** ^*^P < *0.05*, ^**^P < *0.01*, Mean ± SEM.

During the acute phase of IS, glutamate homeostasis is disrupted, and glutamate levels rise above physiological concentrations in the extracellular fluid. This pathological situation leads to an overstimulation of glutamate receptors, followed by the excitotoxic death of neurons. However, the EAAT2 protein, which is mainly expressed in astrocytes, is responsible for 90% of total glutamate uptake to prevent neuronal excitotoxicity [48]. Our previous studies have shown that the expression of EAAT2 was downregulated in YAP^-/-^ astrocytes in vitro and in vivo [23]. Therefore, we next examined whether the increase of excitotoxic death of neurons was due to downregulation of EAAT2 in YAP^GFAP^-CKO mice after IS. As expected, the EAAT2 expression in the infarct area of IS mice was significantly increased compared to the Sham mice, while the expression of EAAT2 in the YAP^GFAP^-CKO mice was decreased in both Sham and IS groups (Fig. 6A, B). Double immunostaining further confirmed that EAAT2 expression was reduced in GFAP^+^ astrocytes of YAP^GFAP^-CKO mice in both Sham and IS groups (Fig. 6D, E).

How does YAP regulate EAAT2 expression in astrocytes? Previous studies have shown that β-catenin is a positive regulator of EAAT2 [23]. Therefore, we next examined whether YAP regulated EAAT2 expression in astrocytes through Wnt/β-catenin signaling. Western blot results showed that the expression level of β-catenin in the cortex of YAP^GFAP^-CKO mice was significantly reduced in both Sham and IS groups (Fig. 6A, C). Double immunostaining of β-catenin and GFAP further confirmed that β-catenin expression was decreased in astrocytes of YAP^GFAP^-CKO mice, compared with that in YAP^f/f^ mice after IS (Fig. 6F, G). These results suggested that the expression of EAAT2 was downregulated in the cortical astrocytes of YAP^GFAP^-CKO mice after IS, which might be due to downregulation of β-catenin signaling.

### Activation of EAAT2 partially restored the deficits such as neuronal death and impairment of behavioral recovery in YAP^GFAP^-CKO mice after IS

**Fig. 7:**
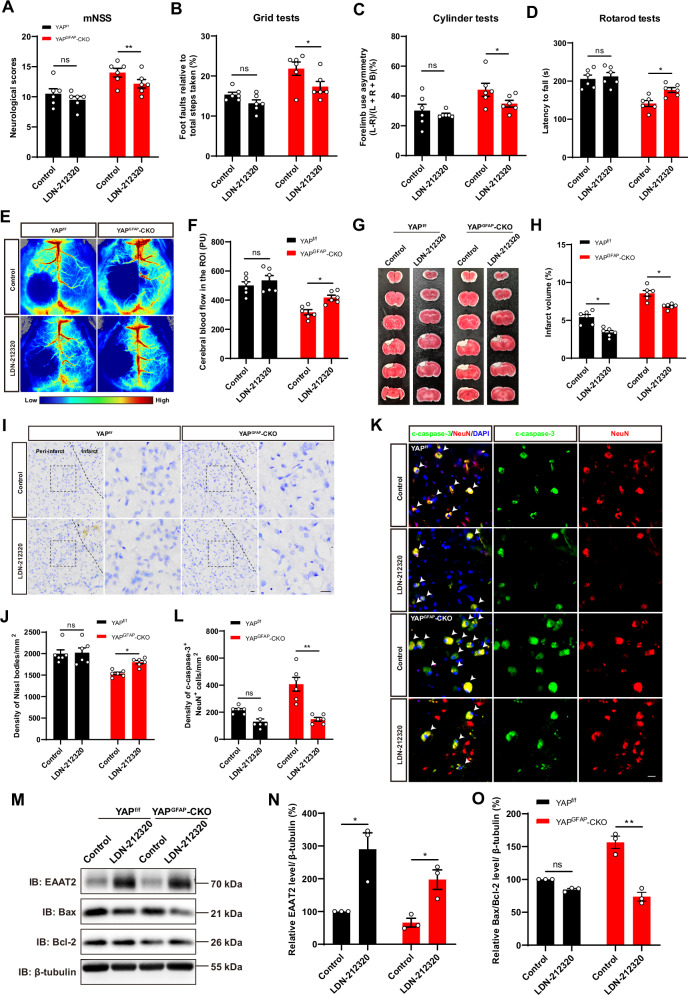
Activation of EAAT2 partially restored the deficits such as neuronal death and impairment of behavioral recovery in YAP^GFAP^-CKO mice after IS. **A–D** Behavioral analysis of YAP^f/f^ and YAP^GFAP^-CKO mice treated with or without LDN-212320 at 3^rd^ day after IS by mNSS (**A**), grid tests (**B**), cylinder tests (**C**) and rotarod tests (**D**) (*n* = 6 mice per group). **E** Representative images of laser scatter imaging of CBF in the infarcted region of YAP^f/f^ and YAP^GFAP^-CKO mice at 3^rd^ day after IS treated with or without LDN-212320. **F** Quantitative analysis of CBF as shown in (**E**) (*n* = 6 mice per group). **G** Representative images of TTC staining analysis of brain tissue from YAP^f/f^ and YAP^GFAP^-CKO mice at 3^rd^ day after IS treated with or without LDN-212320. **H** Quantitative analysis of the infarct volume in the mice shown in (**G**) (*n* = 6 mice per group). **I** Representative images of Nissl staining in the cortex of YAP^f/f^ and YAP^GFAP^-CKO mice at 3^rd^ day after IS treated with or without LDN-212320. **J** Quantification of the density of Nissl bodies as shown in (**I**) (*n* = 6 mice per group). **K** Double immunostaining of c-caspase-3 (green) and NeuN (red) in the cortex of YAP^f/f^ and YAP^GFAP^-CKO mice at 3^rd^ day after IS treated with or without LDN-212320. **L** Quantification of the density of apoptotic NeuN^+^ cells as shown in (**K**) (*n* = 6 mice per group). **M** Western blot analysis of EAAT2, Bax and Bcl-2 in the cortex of YAP^f/f^ and YAP^GFAP^-CKO mice treated with or without LDN-212320 at 3^rd^ day after IS. **N**, **O** Quantification of the relative expression level of EAAT2 (**N**) and Bax/Bcl-2 (**O**), as shown in (**M**) (*n* = 3 mice, normalized to YAP^f/f^ control mice). Scale bars: 20 μm. All data were analyzed by two-way ANOVA with Tukey’s multiple-comparison test. n.s. indicated no statistical difference (*P* > *0.05*); ^*^P < *0.05*, ^**^P < *0.01*, Mean ± SEM.

To examine whether upregulation of EAAT2 could rescue neuronal death and motor dysfunction in YAP^GFAP^-CKO mice after IS. LDN-212320, an activator of EAAT2 at translational level was used. To further confirm whether the treatment of LDN-212320 restored the impairment of functional recovery of YAP^GFAP^-CKO mice, we performed functional behavior tests. Indeed, behavioral tests showed that LDN-212320 treatment significantly improved the motor functional recovery of YAP^GFAP^-CKO mice after IS (Fig. 7A–D). Laser scatter imaging showed that blood flow in brain regions was significantly improved in YAP^GFAP^-CKO mice treated with LDN-212320 (Fig. 7E, F). TTC staining showed that YAP^GFAP^-CKO mice treated with LDN-212320 had smaller infarct volumes (Fig. 7G, H). Nissl staining further confirmed that loss of neurons was significantly decreased in the cortex of YAP^GFAP^-CKO mice treated with LDN-212320 (Fig. 7I, J). Meanwhile, immunostaining of c-caspase-3 also showed that LDN-212320 treatment reduced the density of neuronal apoptosis in the cortex of YAP^GFAP^-CKO mice (Fig. 7K, L). Western blot also showed that the EAAT2 was significantly increased in the YAP^GFAP^-CKO mice treated with LDN-212320 (Fig. 7M, N), and the level of Bax/Bcl-2 was decreased in the YAP^GFAP^-CKO mice treated with LDN-212320 (Fig. 7M, O), indicating that LDN-212320 inhibited the neuronal death of YAP^GFAP^-CKO mice by upregulating EAAT2 after IS. Taken together, these results suggested that activation of EAAT2 by agonist LDN-212320 partially restored the deficits such as the neuronal death and impairment of behavioral recovery in YAP^GFAP^-CKO mice after IS.

### Activation of YAP signaling by XMU-MP-1 promoted the neuronal survival and functional recovery by upregulating EAAT2 expression in mice after IS

**Fig. 8:**
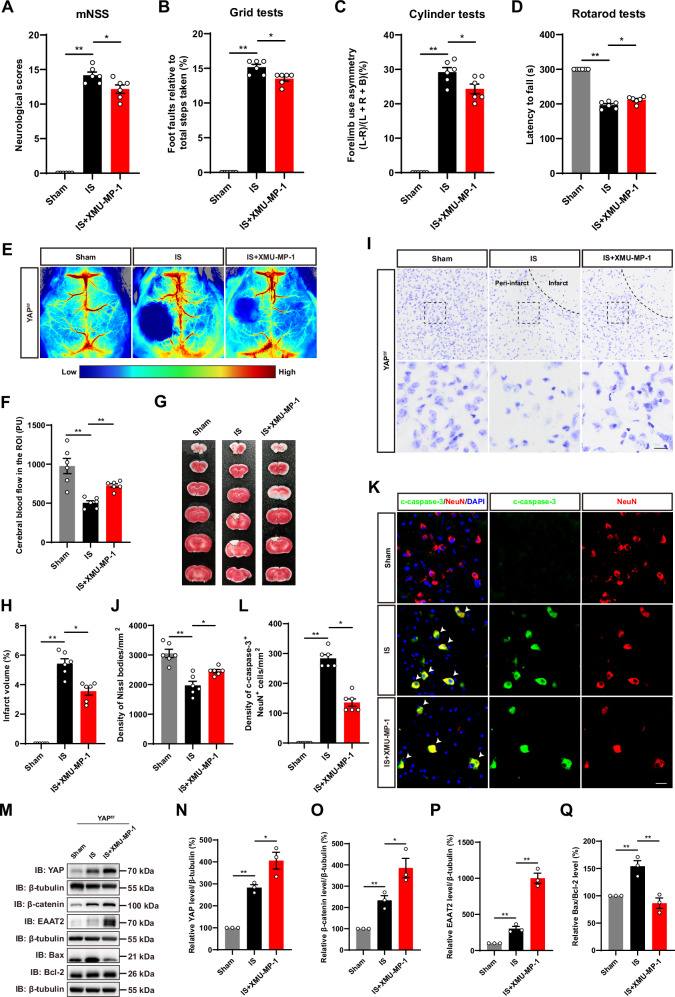
Activation of YAP signaling by XMU-MP-1 promoted the neuronal survival and functional recovery by upregulating EAAT2 expression in mice after IS. **A–D** Behavioral analysis of YAP^f/f^ mice in the Sham group and IS group with or without XMU-MP-1 at 3^rd^ day after IS, including mNSS (**A**), grid tests (**B**), cylinder tests (**C**) and rotarod tests (**D**) (*n* = 6 mice per group). **E** Representative images of laser scatter imaging of CBF in the infarcted region of YAP^f/f^ mice in the Sham group and IS group with or without XMU-MP-1 at 3^rd^ day after IS. **F** Quantitative analysis of CBF as shown in (**E**) (*n* = 6 mice per group). **G** Representative images of TTC staining analysis of YAP^f/f^ mice in the Sham group and IS group with or without XMU-MP-1 brain tissue at 3^rd^ day after IS. **H** Quantitative analysis of the infarct volume in the mice shown in (**G**) (*n* = 6 mice per group). **I** Representative images of Nissl staining in the cortex of YAP^f/f^ mice in the Sham group and IS group with or without XMU-MP-1 at 3^rd^ day after IS. **J** Quantification of the density of Nissl bodies as shown in (**I**) (*n* = 6 mice per group). **K** Double immunostaining of c-caspase-3 (green) and NeuN (red) in the cortex of YAP^f/f^ mice in the Sham group and IS group with or without XMU-MP-1 at 3^rd^ day after IS. **L** Quantification of the density of apoptotic NeuN^+^ cells as shown in (**K**) (*n* = 6 mice per group). **M** Western blot analysis of YAP, β-catenin, EAAT2, Bax, and Bcl-2 in the cortex of YAP^f/f^ mice in the Sham group and IS group with or without XMU-MP-1 at 3^rd^ day after IS. **N–Q** Relative expression levels of YAP (**N**), β-catenin (**O**), EAAT2 (**P**) and Bax/Bcl-2 (**Q**) were quantified as shown in (**M**) (*n* = 3 mice, normalized to Sham group of mice). Scale bar: 20 μm. All data were analyzed by one-way ANOVA with Tukey’s multiple-comparison test. ^*^P < *0.05*, ^**^P < *0.01*, Mean ± SEM.

Previous studies confirmed that XMU-MP-1 selectively inhibited MST1 and MST2, enhanced their downstream YAP activation, with functional recovery promotion in spinal cord injured mice [28], demyelination prevention and neuroinflammation in EAE mice [49, 50], as well as cognitive function improvement in aged mice and AD model mice [51]. Therefore, we investigated whether XMU-MP-1 could promote the neuronal survival and functional recovery by upregulation of EAAT2 expression after IS in mice. As expected, the behavioral tests showed that XMU-MP-1 indeed significantly improved the motor functional recovery in mice after IS (Fig. 8A–D). Laser scatter imaging showed that blood flow in brain regions was also improved in mice treated with XMU-MP-1 (Fig. 8E, F). TTC staining showed that infarct size was smaller in mice treated with XMU-MP-1 (Fig. 8G, H). Nissl staining further confirmed that neuronal loss in the mouse cortex was significantly reduced after XMU-MP-1 treatment (Fig. 8I, J). Meanwhile, immunostaining of c-caspase-3 also showed that XMU-MP-1 treatment reduced the density of neuronal apoptosis in the mouse cerebral cortex (Fig. 8K, L). Western blot confirmed that XMU-MP-1 treatment activated YAP and upregulated the expression of β-catenin and EAAT2 (Fig. 8M–P), further demonstrating that YAP was a key component in the regulation of EAAT2 expression through β-catenin signaling in astrocytes. In addition, Bax/Bcl-2 level was significantly decreased in XMU-MP-1-treated mice (Fig. 8M, Q), revealing that XMU-MP-1 inhibited neuronal death after IS in mice. Taken together, these results strongly suggested that activation of YAP signaling by XMU-MP-1 promoted the neuronal survival and functional recovery by upregulation of EAAT2 expression after IS in mice.

### The activation of YAP signaling by XMU-MP-1 promoted the astrocytic activation and the gain of stemness in astrocytes induced by IS in mice

**Fig. 9:**
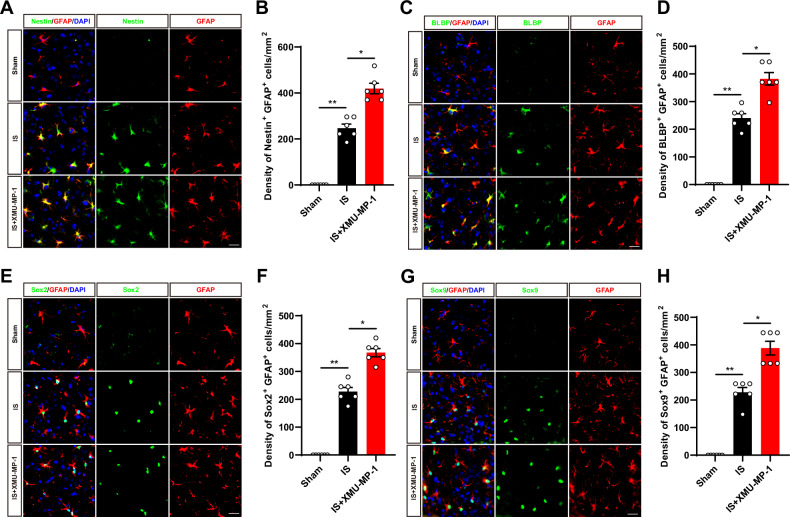
The activation of YAP signaling by XMU-MP-1 promoted the astrocytic activation and the gain of stemness in astrocytes induced by IS in mice. **A** Double immunostaining of Nestin (green) and GFAP (red) in the cortex of YAP^f/f^ mice in the Sham group and IS group with or without XMU-MP-1 at 3^rd^ day after IS. **B** Quantification of the density of Nestin^+^ and GFAP^+^ cells as shown in (**A**) (*n* = 6 mice per group). **C** Double immunostaining of BLBP (green) and GFAP (red) in the cortex of YAP^f/f^ mice in the Sham group and IS group with or without XMU-MP-1 at 3^rd^ day after IS. **D** Quantification of the density of BLBP^+^ and GFAP^+^ cells as shown in (**C**) (*n* = 6 mice per group). **E** Double immunostaining of Sox2 (green) and GFAP (red) in the cortex of YAP^f/f^ mice in the Sham group and IS group with or without XMU-MP-1 at 3^rd^ day after IS. **F** Quantification of the density of Sox2^+^ and GFAP^+^ cells as shown in (**E**) (*n* = 6 mice per group). **G** Double immunostaining of Sox9 (green) and GFAP (red) in the cortex of YAP^f/f^ mice in the Sham group and IS group with or without XMU-MP-1 at 3^rd^ day after IS. **H** Quantification of the density of Sox9^+^ and GFAP^+^ cells as shown in (**G**) (*n* = 6 mice per group). Scale bars: 20 μm. All data were analyzed by one-way ANOVA with Tukey’s multiple-comparison test. ^*^P < *0.05*, ^**^P < *0.01*, Mean ± SEM.

Our above results suggest that YAP deletion in astrocytes inhibits astrocyte activation and attenuates the gain of stemness in astrocytes at the edge of the infarct zone. Thus, we next examined whether activation of YAP promoted astrocytic activation and the gain of stemness in astrocytes induced by IS in mice. We utilized XMU-MP-1 (MST1/2 inhibitor) to inhibit the Hippo signaling pathway to activate YAP in IS mice. As expected, double immunofluorescence showed that the density of GFAP and Nestin double-positive cells was significantly increased in the peri-infarct area of IS mice treated with XMU-MP-1 (Fig. 9A, B). Meanwhile, the density of GFAP and BLBP (a marker of astrocyte transition to the intermediate state [45]) double-positive cells was also significantly increased in the peri-infarct area of IS mice treated with XMU-MP-1 (Fig. 9C, D). In addition, Sox2 and Sox9 acted as major transcription factors for the induction and maintenance of neural stem cells [46, 47]. The density of GFAP and Sox2 double-positive astrocytes was significantly increased in the infarct margins of IS mice treated with XMU-MP-1 (Fig. 9E, F), and following co-staining with Sox9, we observed a similar increase in the density of Sox9 and GFAP double-positive astrocytes after XMU-MP-1 treatment (Fig. 9G, H). Taken together, these results strongly suggested that activation of YAP signaling promoted the astrocytic activation and the gain of stemness in astrocytes induced by IS in mice.

## Discussion

**Fig. 10:**
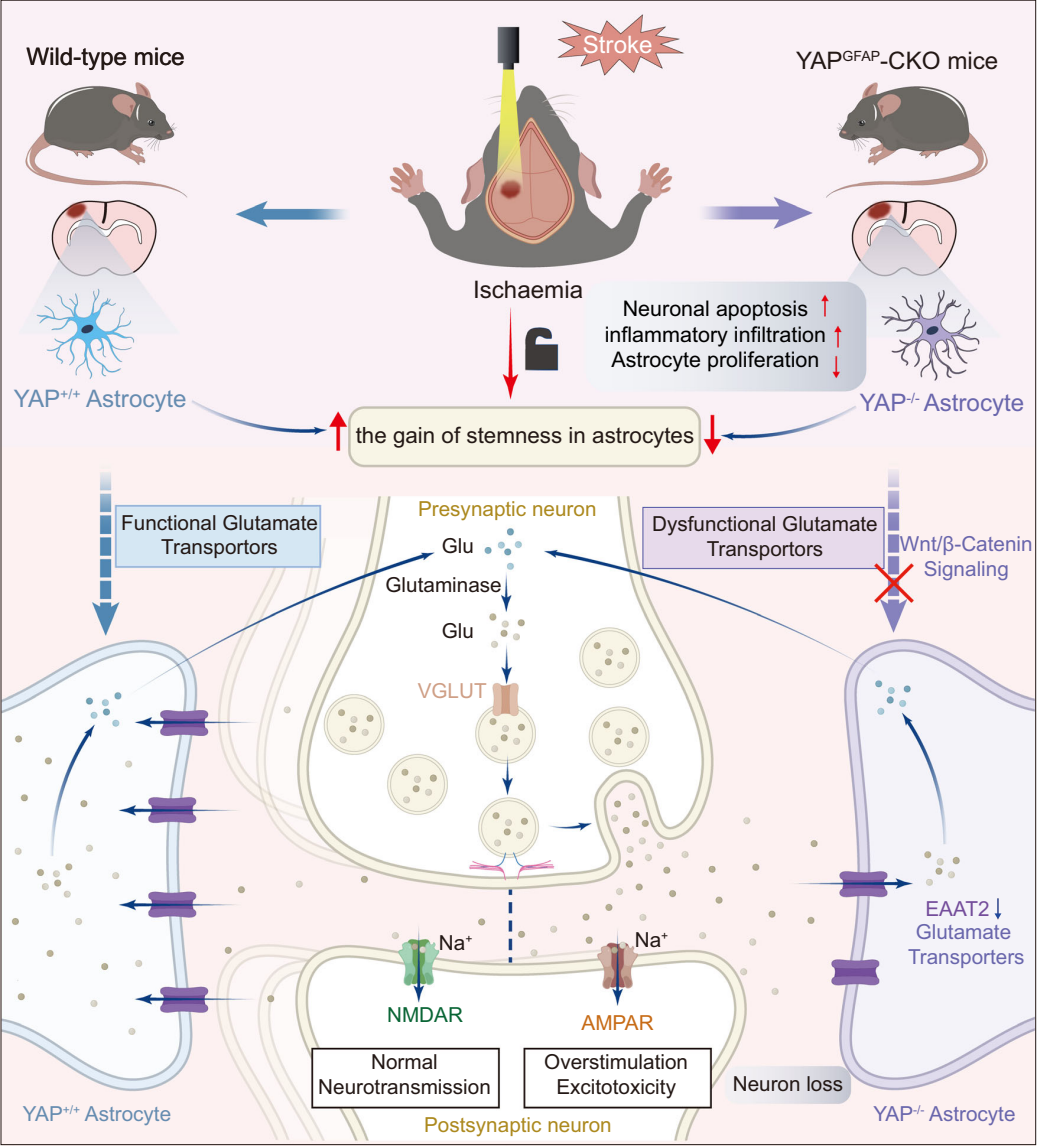
Working model of astrocytic YAP signaling in ischemic stroke mice. After IS, YAP is upregulated and activated in astrocytes. Knockout YAP in astrocytes aggravates the IS with inhibition of the functional behavioral recovery, larger injury area, more apoptotic neurons and more inflammatory infiltration, which may be caused by impairment of the reduction of astrocytic proliferation, inhibition of astrocytic activation and stemness after IS, and promoting the glutamate excitotoxicity through downregulation of β-catenin/EAAT2 signaling.

Here, we revealed that the deletion of astrocyte-specific YAP deteriorated the neuronal death during IS, implying the neuroprotective effect of astrocytic YAP signaling in IS for the first time. These findings may have significant clinical treatment of IS for the following reasons (see Fig. 10 working model). Firstly, we found that exposure to IS activated and upregulation of YAP in astrocyte early after the ischemic occlusion, suggesting that YAP signaling might play an important role in IS. Secondly, we performed genetic experiments and showed that YAP deletion in astrocyte decreased cerebral blood flow, increased the infarct volume, prevented the behavioral recovery, aggravated neuronal death, increased inflammatory infiltration, and impaired the formation of glial scars by inhibiting astrocyte proliferation, strongly supporting the neuroprotective effect of astrocytic YAP signaling in IS. Interestingly, we found that YAP signaling also controlled the gain of stemness in astrocytes induced by IS in mice. Thirdly, we found that the therapeutic potential of YAP-EAAT2 signaling for treating IS. LDN-212320 indeed restored the deficits such as neuronal death and impairments of behavioral recovery in YAP^GFAP^-CKO mice after IS, and XMU-MP-1 activated YAP and, restoring the deficits of neuronal death and impairing behavioral recovery after IS through β-catenin/EAAT2 pathway in wild-type mice. Furthermore, activation of YAP by XMU-MP-1 promoted astrocytic activation and the gain of stemness in astrocytes induced by IS in mice. Altogether, these results reveal the neuroprotective role of YAP-EAAT2 in IS and suggest a previously unidentified role for YAP in regulating poststroke neuronal death and provide a drug target for the therapeutic intervention.

In our previous studies, we have demonstrated that YAP is highly expressed in astrocytes but not in neurons or microglia, both under physiological conditions and in various disease models except IS [18, 28, 49, 50]. YAP has reported to be accumulated in the nucleus of astrocytes with a significant increase in expression during the onset of IS, and activation of the nuclear expression of YAP attenuates brain damage in IS [22]. Our results showed that following IS, YAP expression peaked at 3^rd^ day, paralleling a significant nuclear localization with GFAP-positive astrocytes.

In addition, numerous studies have indicated that reactive gliosis and glial scar formation can serve as neuroprotective mechanisms at certain stages and regulate neuroplasticity, including neurogenesis, synaptic control, as well as maintaining CNS homeostasis and limiting neuroinflammation [52–54]. Initially, scarring by reactive astrocytes was thought to inhibit axonal regeneration, and it was assumed that such scarring was a major obstacle to functional recovery after CNS injury or disease. Reactive astrocytes have been later shown to protect CNS cells and tissues in various ways. After stroke, glial scarring can limit the spread of inflammatory cells from the injured area to healthy CNS parenchyma [55]. It has been previously shown that conditional knockout of YAP in astrocytes significantly inhibits astrocyte proliferation and impairs glial scar formation, resulting in inhibiting the neurodegeneration after spinal cord injury [28]. In our acute stroke model, conditional knockout of YAP in astrocytes also inhibited astrocyte proliferation, which in turn impeded glial scar formation and led to disease exacerbation. This is consistent with previous views of the protective effect of glial scarring on healthy CNS parenchyma at the margin of injury after stroke. However, in the late recovery phase after stroke, glial scarring may impede axonal regeneration and functional recovery of patients [53, 56]. Therefore, in IS, glial scarring plays beneficial and harmful roles with different disease processes.

Under physiological conditions, mature astrocytic cells are partially specialized precursors that are functionally adapted to the local neuronal microenvironment. Under pathological or injury-induced conditions, intrinsic factors and environmental cues allow astrocytes to re-enter the cell cycle and differentiate into other neuronal cell types [7]. It has been shown that specialized astrocytes in the subventricular zone (SVZ) and hippocampal dentate gyrus of adult mice are pluripotent neural stem cells (NSCs) that continuously generate new neurons [57]. In the medial striatum, the stroke induces the parental astrocytes to activate the neurogenic program through the repression of the Notch signaling pathway. This neurogenic process involves transit-amplifying divisions instead of direct trans-differentiation [58–60]. It is noteworthy that some of the reactive astrocytes in the glial proliferative phase are highly proliferative following lesions. In terms of transcriptomic features, including increased MIK67 expression, tumor-associated “reactive” astrocytes overlap significantly with immature proliferative astrocytes [61]. It has been suggested that after ischemic brain injury, reactive astrocytes may participate in neurogenesis by transforming into neurons and promote brain repair after IS [8, 62]. Reactive astrocytes around the site of injury have been shown to have stem cell-like properties similar to neural precursor cells, including self-renewal and pluripotency [63]. Recent studies have shown DNA methylation controls stemness of astrocytes in health and ischemia. Ischemia injury-induced neurogenesis in the striatum depends on the de novo methyltransferase DNMT3A [11]. Interestingly, DNMT3A recruited by YAP/TAZ guides DNA methylation to drive gallbladder cancer metastasis [24], and YAP signaling contributes to DNA methylation remodeling upon mouse embryonic stem cell differentiation [25]. Consistent with these previous studies, we found that YAP deletion in astrocytes suppressed the activation of astrocytes and diminished the gain of stemness in astrocytes at the edge of the infarct zone at 3^rd^ day after IS, and then hindered brain repair after ischemia. Whereas activation of YAP using XMU-MP-1 promotes astrocyte activation and gain of stemness in astrocytes after IS in mice. In future, further studies need to be carried out to test whether YAP promotes the gain of stemness in astrocytes through DNA methylation after ischemia.

In addition, our previous studies found that YAP deficiency in astrocytes reduces the expression of the glutamate transporter EAAT2, which helps to maintain glutamate homeostasis in CNS. Under normal conditions, astrocytic glutamate transporters are indirectly involved in the termination of synaptic transmission by regulating glutamate availability at postsynaptic neuronal receptors [64]. In addition, astrocytes regulate neurotransmitter release by controlling the amount of glutamate reaching presynaptic receptors, finally glutamate uptake by astrocytes protects neurons from hyperexcitability and subsequent excitotoxic damage by maintaining extracellular glutamate concentrations under neurotoxic levels [5]. Extracellular glutamate concentration showed transient elevations immediately after an ischemic attack (within 30 min). Although glutamate-induced excitotoxicity is a crucial component of the initial neuronal death of IS, all tested anti-glutamatergic drugs have failed in clinical trials [65]. The main reason is the rapid and early increase in extracellular glutamate post-IS, while the drugs are administrated several hours after the event. Another approach is to increase the uptake of glutamate by glial cells and increase the clearance of glutamate from the synaptic gap to inhibit neuronal excitotoxicity [66]. Glutamate uptake by glutamate transporters plays a major role, especially EAAT2 which is predominantly located in astrocyte, has been reported to account for 80–90% of glutamate uptake in CNS [67]. Our results showed YAP deletion in astrocytes decreased EAAT2, which supported the idea that decrease in YAP impeded the expression of EAAT2 that might lead to a decrease in glutamate uptake and subsequent excitotoxicity in IS.

We used PTS model of IS in this study. The MCAO model, extensively utilized in numerous previous studies, posed risks of vessel rupture, hemorrhage, and high mortality attributed to large infarcts. Moreover, it frequently led to hypothalamic injury, which was rare in human stroke cases. Conversely, the photochemical embolization model has emerged as a more specific approach for studying stroke in recent years. Advantages include predictable and unambiguous localization of ischemic lesions through targeted laser beams to predetermined brain regions, determination of lesion extent by light intensity and duration, facilitating easy administration, high reproducibility of lesions owing to stereotactic precision of irradiation and continuous light intensity monitoring, low invasiveness and minimal surgical intervention, resulting in low animal mortality (<10%), prolonged sensory-motor deficits and enhanced feasibility for long-term studies of behavioral deficits and recovery post-stroke [68].

Currently, no effective adjunct therapies can limit neuronal damage in patients with IS. A particular strength of the study is that we demonstrate that targeting YAP-EAAT2 improves outcomes, which will be a translational potential. In this study, we only examined the stroke outcomes at the acute stages of stroke in young mice. It is important to also evaluate the effect of the astrocytic YAP-EAAT2 signaling at the chronic phase of IS and in elder mice, which is underway in our lab. In addition, because the prevalence of IS is significantly higher in males than in females [69] and estrogen is neuroprotective after IS [70], we investigated the effects of YAP signaling on IS only in male mice. There is now evidence that males and females may respond differently to stroke and therapeutic interventions. Our preliminary results found that there were no significant differences in functional behavior recovery between female and male YAP^GFAP^-CKO mice after IS (data not shown), so in the future we will verify the generalizability of the results in female model.

In conclusion, our data demonstrate that astrocytic YAP-EAAT2 signaling is critical for the neuroprotective of post-IS. The effect is likely achieved through the glutamate uptake to inhibit neuronal death from excitotoxicity, the promotion of glial scar formation and gain of stemness in astrocytes after IS (Fig. 10 working model). Our study provides a novel drug target for IS treatment.

## Supplementary information

Supplement figure 1

Supplementary materials

## Data Availability

All data generated or analysed during this study are included in this published article [and its supplementary information files].
