# Supplementary material for: Astrocytic YAP prevents the glutamate neurotoxicity by upregulation of EAAT2 expression and promotes the gain of stemness in astrocytes in ischemic stroke mice
Source: Cell Death Dis. 2025 Jul 30;16(1):577. doi: 10.1038/s41419-025-07806-7 (PMC12310981; doi:10.1038/s41419-025-07806-7)
Supplement: Supplementary file 2 — Supplementary materials [file 41419_2025_7806_MOESM2_ESM.docx]

**Supplementary materials**

**Figure S1 TCC staining of brain sections in IS mice, normal brain development and motor function in YAP^GFAP^-CKO mice.**

(**A**) Representative TTC-stained brain sections of wild-type mice at 3^rd^ day after IS. (**B**) Quantitative analysis of cerebral infarct volume in mice as shown in (**A**) (n=6 mice per group). (**C**) Representative images of body size of 6-week-old YAP^f/f^ and YAP^GFAP^-CKO mice. (**D**) Genotyping of 6-week-old YAP^f/f^ and YAP^GFAP^-CKO mice. (**E**) Western blot analysis of YAP expression in the cortex, hippocampus, cerebellum and spinal cord of 6-week-old YAP^f/f^ and YAP^GFAP^-CKO mice. (**F**) Quantitative analysis of the relative YAP level as shown in (**E**) (n=3 mice per group, normalized to YAP^f/f^ control mice). (**G**) Double immunostaining of YAP (green) and GFAP (red) in the cortex of YAP^f/f^ and YAP^GFAP^-CKO mice. (**H-K**) Behavioral analysis of 6-week-old YAP^f/f^ and YAP^GFAP^-CKO mice by mNSS (**H**), grid tests (**I**), cylinder tests (**J**) and rotarod tests (**K**) (n=6 mice per group). Scale bars: 20 μm. Data were analyzed by unpaired *t*-test analysis (**B, F, H-K**). n.s. indicated no statistical difference (*P>0.05*); *^**^P<0.01*, Mean ± SEM.
